# Versatile Single-Element Ultrasound Imaging Platform using a Water-Proofed MEMS Scanner for Animals and Humans

**DOI:** 10.1038/s41598-020-63529-z

**Published:** 2020-04-16

**Authors:** Seongwook Choi, Jin Young Kim, Hae Gyun Lim, Jin Woo Baik, Hyung Ham Kim, Chulhong Kim

**Affiliations:** Department of Creative IT Engineering, Electrical Engineering, and Mechanical Engineering, Pohang University of Science and Technology (POSTECH), Pohang, 37673 Republic of Korea

**Keywords:** Ultrasound, 3-D reconstruction, Biomedical engineering

## Abstract

Single-element transducer based ultrasound (US) imaging offers a compact and affordable solution for high-frequency preclinical and clinical imaging because of its low cost, low complexity, and high spatial resolution compared to array-based US imaging. To achieve B-mode imaging, conventional approaches adapt mechanical linear or sector scanning methods. However, due to its low scanning speed, mechanical linear scanning cannot achieve acceptable temporal resolution for real-time imaging, and the sector scanning method requires specialized low-load transducers that are small and lightweight. Here, we present a novel single-element US imaging system based on an acoustic mirror scanning method. Instead of physically moving the US transducer, the acoustic path is quickly steered by a water-proofed microelectromechanical (MEMS) scanner, achieving real-time imaging. Taking advantage of the low-cost and compact MEMS scanner, we implemented both a tabletop system for *in vivo* small animal imaging and a handheld system for *in vivo* human imaging. Notably, in combination with mechanical raster scanning, we could acquire the volumetric US images in live animals. This versatile US imaging system can be potentially used for various preclinical and clinical applications, including echocardiography, ophthalmic imaging, and ultrasound-guided catheterization.

## Introduction

Ultrasound (US) imaging is widely used to obtain *in vivo* structural, functional, and molecular information based on the acoustic properties of tissue and other anatomical components in animals and humans. Typical US imaging systems employ multi-element US array transducers and multi-channel US transmitting and receiving electronics. However, this approach makes the US system relatively complex, bulky, and expensive. Particularly, the manufacturing process is more complicated and costly for high frequency US array transducers, which hinders the wide-spread use of high resolution US imaging. Nevertheless, the use of high frequency US imaging has become increasingly desirable in various preclinical (echocardiography, oncology, ophthalmology) and clinical (ultrasound-guided catheterization, musculoskeletal imaging, ophthalmology) applications^1–8^.

Temporal resolution is a major limitation of single-element based US imaging. Although this modality requires only single-channel electronics and a single-element transducer, which are relatively simple, compact, and cost-effective, its real-time imaging ability is less than that of array-based US imaging. Scanning is required to achieve cross-sectional B-mode imaging with a single-element transducer, and both mechanical linear and mechanical sector scanning have been intensively explored. Mechanical linear scanning translates a single-element transducer mechanically with a motorized stage, but the achievable frame rate is limited by the low scanning speed. For faster imaging, mechanical sector scanning rotates a single-element transducer within a given angular range about a fixed point^1,2,9^. Although it can achieve real-time US imaging, sector scanning requires customized US transducers that are compact and lightweight to reduce the inertial load of the rotating system.

Spatial resolution, on the other hand, is an important advantage of single element based US imaging over array-based imaging. A single element transducer with a central circular aperture has a larger effective area than an array transducer with a rectangular aperture. Given the same operating frequency and the same aperture width, since a spherical aperture has a larger area than a rectangular aperture, a single element transducer gives a more tightly focused beam with a narrower beam width. Therefore, the lateral resolution of the images generated by a single element transducer is better than those generated by an array transducer.

In this paper, to overcome the limit of temporal resolution while realizing superior spatial resolution, we propose a novel single-element US imaging system based on an acoustic mirror scanning method. This approach steers the acoustic path more quickly than physically moving the US transducer. Thus, unlike mechanical sector scanning, the mirror scanning does not highly depend on the physical dimensions and weight of the single-element transducer. To steer the acoustic path, we employed a water-proofed microelectromechanical system (MEMS), a design which has been recently employed in photoacoustic microscopy and optical coherent tomography^10–24^. Some studies have been performed US imaging on phantoms *in vitro*, but not *in vivo*^25,26^.

This water-proofed MEMS scanner is relatively cheap and the fabrication process is also relatively simple. Further, the acoustic steering speed is fast enough to perform real-time B-mode imaging. In addition, this miniaturized and simple MEMS scanner can be easily deployed on various platforms. We developed two types of single-element US imaging systems: tabletop (TT) and handheld (HH) systems. The tabletop system, equipped with motorized stages, was developed mainly for wide field-of-view (FOV) *in vivo* volumetric animal imaging. In practice, the 3D US imaging system overcomes operator dependence which typically exists in 2D US imaging^25,27–30^. To demonstrate the system, we acquired 2D and 3D US images of mouse hearts and throats. The handheld system was developed mainly for *in vivo* 2D B-mode human imaging. It successfully provided B-mode images of the radial artery and vein of a human volunteer including image processing in real-time without GPU parallel computing (i.e., a B-mode imaging rate of 40 Hz).

## Results

### Single-element ultrasound imaging platform using a water-proofed MEMS scanner

**Figure 1:**
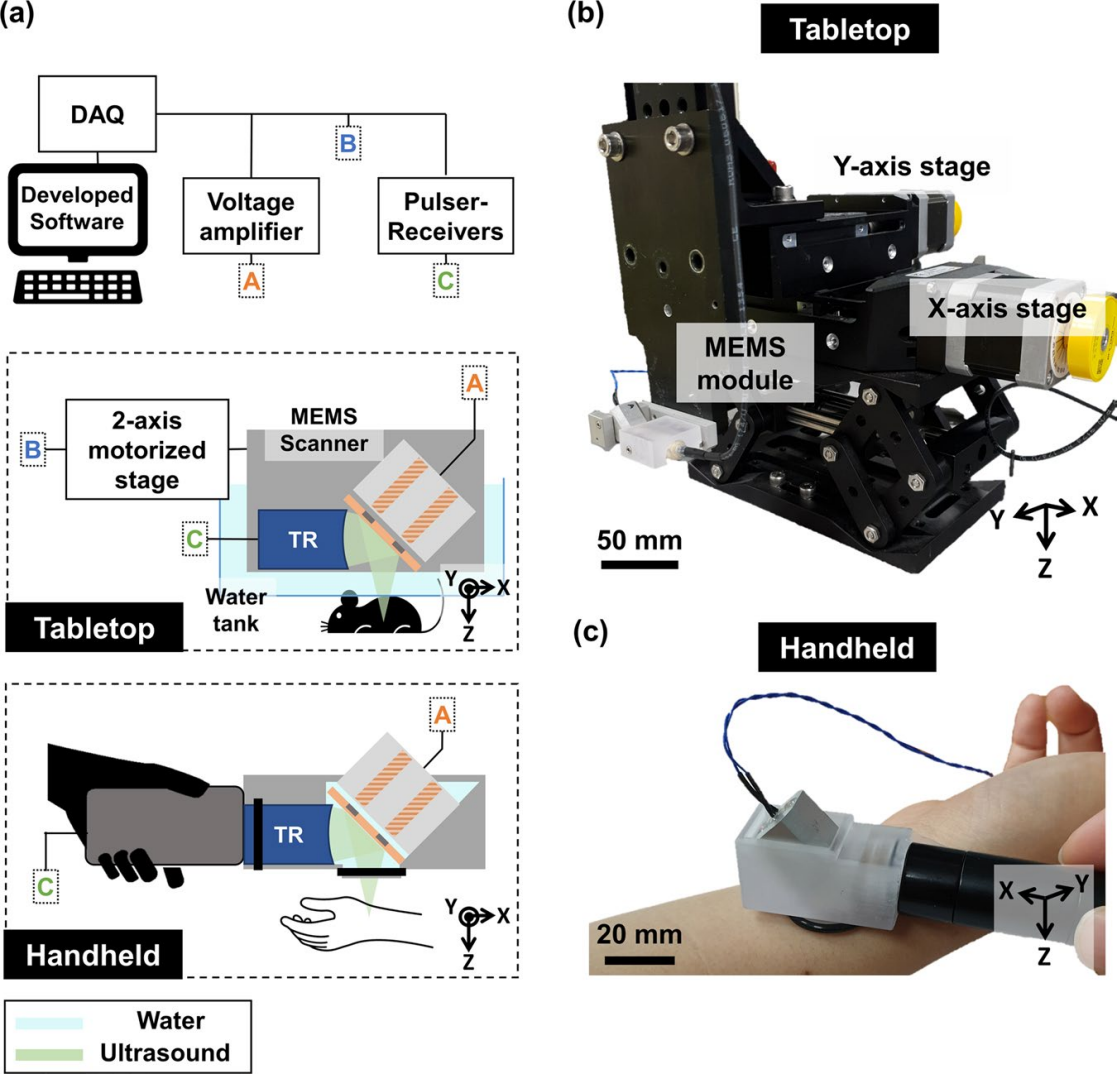
(**a**) Schematic of tabletop and handheld version of the single-element ultrasound (US) imaging system using a water-proofed MEMS scanner (MEMS-US). Photographs of (**b**) the tabletop 3D MEMS-US system for small animals and (**c**) the handheld 2D MEMS-US system for humans.

We developed both tabletop and handheld imaging systems (Fig. 1(a)). The tabletop-mode US imaging system using the MEMS scanner (MEMS-US-TT) is equipped with two single-axis motorized stages (PLS-85, Physik Instrumente GmbH & Co. KG, Germany) (Fig. 1(b)). To prevent water leakage and reduce acoustic attenuation, the water tank is manufactured with an open bottom sealed with removable plastic film that conforms closely to the target’s contours. The much smaller handheld-mode US imaging system (MEMS-US-HH) measures 50 mm × 20 mm × 20 mm along the X, Y, and Z axes, respectively, and its total weight, including the MEMS scanner and the US transducer, is below 60 g (Fig. 1(c)). The fixture for holding the MEMS scanner and the US transducer is also manufactured by 3D printing, and rubber O-rings (NPF10 and NPF16, MiSUMi, USA) form watertight seals.

The system workflow is as follows: first, the input parameters (e.g., the US pulse repetition frequency (PRF), data size, number of steps in the volumetric imaging, and step size) are entered into a graphical user interface (GUI) developed in LabVIEW. We used a PRF of 10 kHz and a B-mode data size of 125 × 2048 double floating point pixels, which depends on the PRF of the pulser-receiver and MEMS scanning while the axial pixel size does on the sampling rate of a digitizer. Second, a data acquisition (DAQ) board (NI-PCIe-6321, National Instruments, USA) generates the analog output to steer the acoustic mirror and the digital output to trigger the entire system. This analog output is amplified by both an operation amplifier (TCA0372, ON Semiconductor, USA) and isolated DC-to-DC converter (NDS6D2415C, Murata Power Solutions, Japan). A pulser-receiver (5073PR, Olympus NDT Inc., USA) transmits pulses followed by the external trigger generated in the DAQ. Then, a high-speed digitizer (ATS9350, Alazar Technologies Inc., Canada) receives the reflected US signal at a sampling rate of 100 MS/s and with a dynamic range of 400 mV. The GUI program performs image processing steps on the raw data.

In the image processing steps, the DC components of each A-line of raw data are initially removed by averaging them, and a band-pass filter is employed to remove noise. Then, time gain compensation reduces the differences in the intensities of the signals with depth, caused by tissue attenuation. Frequency demodulation and log compression follow, and finally scan conversion is employed to create a B-mode image of the actual scanned shape. Finally, the developed LabVIEW program shows real-time B-mode images.

In 3D volumetric imaging, we define local and global volumetric data. Local volumetric data is obtained with the single-axis MEMS scanner along the X-axis and the single-axis motorized stage along the Y-axis, which scan perpendicular to each other. To get a large FOV image for global volumetric data, this step is repeated with an additional single-axis motorized stage along the X-axis. These local volumetric data are merged to generate one global mosaic dataset in MATLAB, using a Gaussian window for apodization to reduce seams. We use our own volume rendering program developed using the volume ray-casting algorithm, and parallel computing with a graphics processing unit (GPU). The detailed specifications of the MEMS-US system are summarized in Table 1 (Supplementary Fig. S1).

**Table 1:** Specifications of the MEMS-US System.

Transducer center frequency	16.7 MHz
Transducer aperture size	9 mm
Transducer focal length	21.8 mm
Transducer fractional bandwidth	51.7%
Transducer depth of focus	4.1 mm
B-scan frame rate	40 Hz
Lateral resolution	203 μm
Axial resolution	100 μm

### *In vitro* phantom imaging

**Figure 2:**
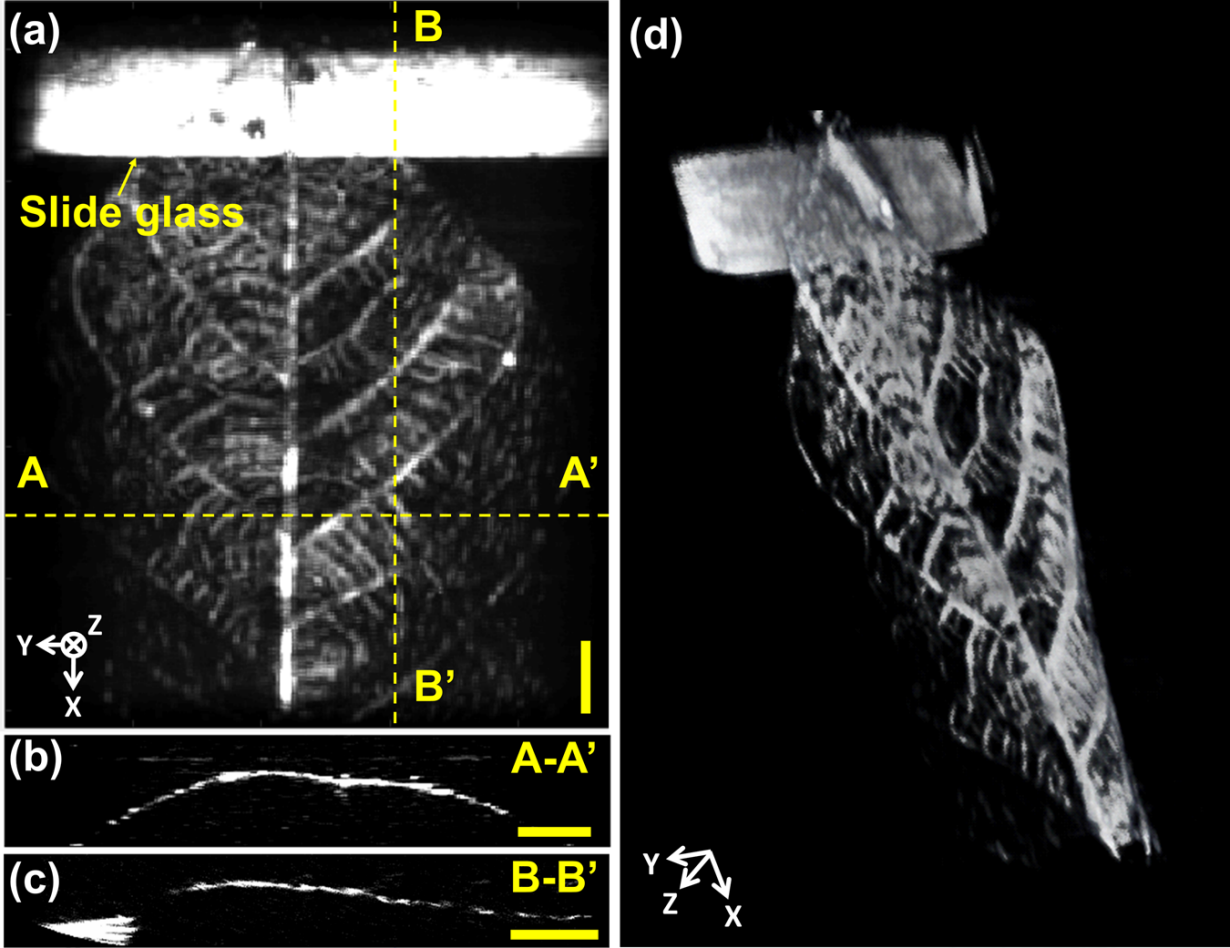
3D volumetric ultrasound imaging of a leaf skeleton. (**a**) Maximum intensity projection (MIP) image. (**b**) Global cross-sectional B-mode images along (**b**) the line A-A’ and (**c**) the line B-B’. (**d**) 3D rendered image. Supplementary Movie S1. All scale bars are 3 mm.

To demonstrate 3D imaging, we used the MEMS-US-TT system to image a leaf skeleton phantom whose stem end was held in place on the bottom of the water tank beneath the weight of a glass slide. When the water tank was filled, the buoyant main body of the leaf skeleton floated slightly up, giving the skeleton a three-dimensional structure. We obtained six local volumetric datasets of the leaf skeleton phantom to form a global volumetric image. Then, the maximum intensity projection (MIP) image was processed in MATLAB (Fig. 2(a)). Figure 2(b,c) show global cross-sectional B-mode images of the leaf skeleton cut along the lines A-A’ and B-B’, respectively. As one can see, the seams between the local volumetric datasets are clearly removed. The curved shape of the leaf skeleton is shown well in the 3D volume rendered images (Fig. 2(d) and Supplementary Movie S1)^31^. These results demonstrate that the imaging method and the image reconstruction algorithm used in this study are suitable for *in vivo* experiments.

### *In vivo* small animal imaging

**Figure 3:**
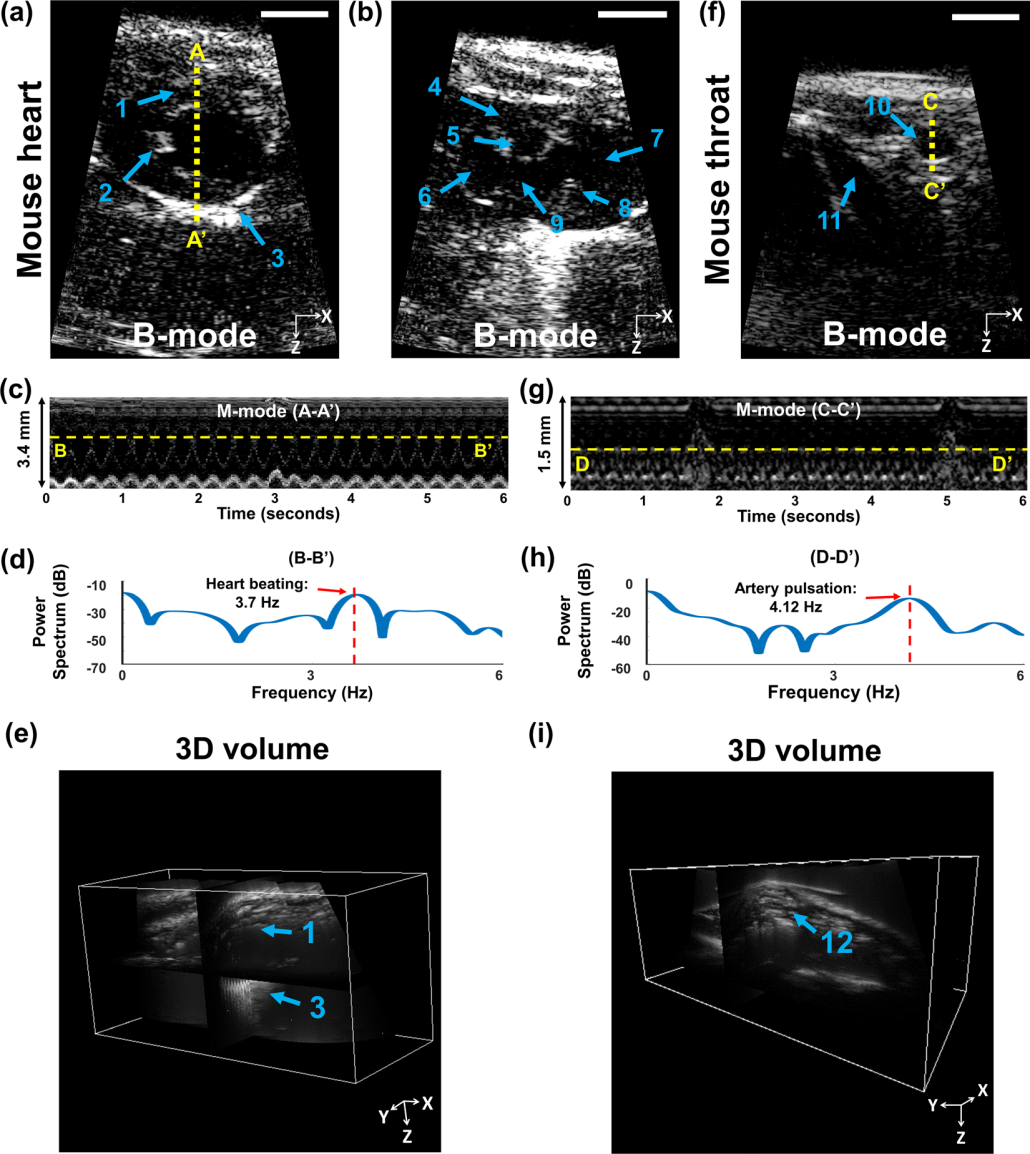
*In vivo* ultrasound imaging of a mouse with the MEMS-US-TT (tabletop) system. (**a**,**b**) B-mode images of the mouse heart from two different angles. (**c**) M-mode image of the line A-A’. (**d**) Power spectrum of the line B-B’. (**e**) 3D volume image of the mouse heart. (**f**) B-mode image of the mouse throat. (**g**) M-mode image of the line C-C’. (**h**) Power spectrum of the line D-D’. (**i**) 3D volume image of the mouse throat. All scale bars are 3 mm. 1, left ventricle (LV) anterior wall; 2, LV papillary muscle; 3, LV posterior wall; 4, left atrium; 5, aortic valve; 6, pulmonary artery; 7, right atrium; 8, pulmonary valve; 9, right ventricular outflow tract; 10, aortic arch; 11, jugular vein; 12, carotid artery.

We investigated the *in vivo* imaging capability of the MEMS-US-TT system by acquiring B-mode and M-mode images of a mouse heart at 40 frames per second (fps). Figure 3(a,b) are snapshots of real-time B-mode imaging acquired at two different angles. Figure 3(a) shows the left ventricle (LV) anterior wall (AW), labeled 1; the LV papillary muscle (PM), labeled 2; and the LV posterior wall (PW), labeled 3. Figure 3(b) shows the left atrium (4), the aortic valve (5), the pulmonary artery (6), the right atrium (7), the pulmonary valve (8), and the right ventricular outflow tract (9). The inner structures are well shown, compared with the image acquired by an array US transducer^32^.

We then successfully visualized the dynamics of a beating mouse heart through M-mode imaging. As shown in Fig. 3(c), the contraction and expansion of the LVAW and the LVPW are appear clearly in a repetitive pattern, and one irregular pattern at 3 seconds is also apparent, showing respiration. To calculate the heartbeat rate, we analyzed the power spectrum along the line B-B’ in the M-mode image, and found that the heartbeat repeated with a frequency of 3.7 Hz (Fig. 3(d)). A mouse’s heart typically beats at between 310 and 840 beats per minute (bpm). In this experiment, however, anesthesia decreased the heartbeat to 222 bpm.

Next, we obtained a 3D image of the entire mouse heart by performing global mosaic imaging, with the results shown in Fig. 3(e) and Supplementary Movie S2. The 3D volume rendered image also shows the LVAW and the LVPW. Following the same imaging procedures, we also obtained B-mode images of the mouse throat region as seen in Fig. 3(f). The aortic arch (10) and the jugular vein (11) are identifiable. The aortic arch was pulsating in response to the heart’s pumping, so its M-mode image has a repetitive pattern (Fig. 3(g)). The irregularities at 1.8 and 5 seconds indicate respirations. We could verify the pulsation period of the aortic arch as 4.12 Hz, which corresponds to 247 bpm, through power spectrum analysis of the line D-D’ in the M-mode image (Fig. 3(h)). Similarly, we acquired a 3D volumetric image of the throat, in which we could identify the carotid artery, in an YZ plane cross-section (Fig. 3(i) and Supplementary Movie S3).

### *In vivo* human imaging

**Figure 4:**
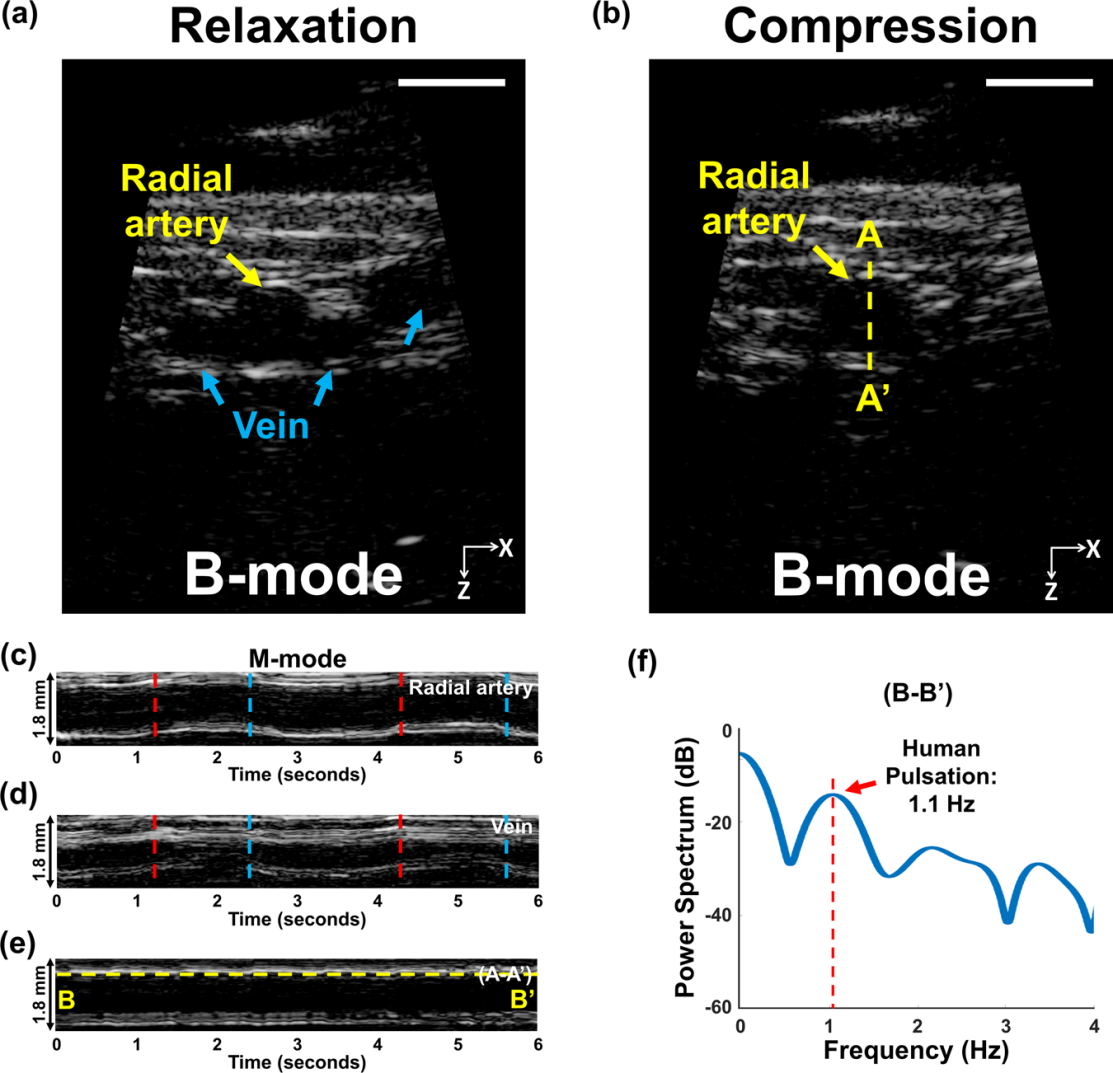
*In vivo* ultrasound imaging of the wrist of a human volunteer, obtained with the MEMS-US-HH (handheld) system. Cross-sectional B-mode images of the human wrist (**a**) in relaxation and (**b**) in compression. M-mode images of (**c**) the radial artery, (**d**) the vein, and (**e**) the line A-A’. (**f**) The power spectrum of the line B-B’. All scale bars are 3 mm. Red and blue lines in (**c**,**d**) indicate compression and relaxation, respectively.

We explored the *in vivo* imaging ability of the MEMS-US-HH system by applying it to a human volunteer’s wrist. Figure 4(a) is a cross-sectional B-mode image of the volunteer’s wrist in a relaxed state without compression, showing a radial artery and veins. Once we compressed the wrist with the MEMS-US-HH system, the veins disappeared but the radial artery, relatively speaking, maintained its shape and thickness (Fig. 4(b)). Figure 4(c,d) are M-mode images simultaneously acquired from the radial artery and the vein, respectively. The red and blue lines in Fig. 4(c,d) indicate the beginnings of compression and relaxation, respectively. The thickness of the radial artery is unchanged, although the position is slightly shifted. However, the veins are almost completely blocked during the compression.

When we held the system in the fixed position, we were able to identify the arterial pulsation. Figure 4(e), an M-mode image of the radial artery in a static condition, shows a repetitive pattern generated by the pulsation. We performed a power spectrum analysis on the line B-B’ and could quantify that the radial artery’s pulsation period was 1.1 Hz, which corresponds to 66 bpm (Fig. 4(f)). This rate fits within the normal resting heart rate of between 60 and 100 bpm.

## Discussion

We developed the first *in vivo* 2D and 3D US imaging system with a single-element US transducer and a water-proofed MEMS scanner. In small animal US imaging studies, it is difficult to position and fix a US probe to accurately locate internal structures (e.g., heart, artery, or vein) because a cross-sectional B-mode image is completely disturbed when the angle or position of the US probe is slightly mispositioned. Therefore, the tabletop-mode US imaging system was used in the small animal studies. Because it uses two single-axis motorized stages in combination with the MEMS scanner, the MEMS-US-TT system could precisely image 2D and 3D internal structures in small animals. We were also able to monitor dynamic movements, such as the heart beating and aorta pulsating, because a high frame rate of 40 Hz was achieved by the rapid rotation of the MEMS scanner. Currently, the system faces third problems. First, because of the fixed focal zone, the image quality becomes poor in out-of-focus regions. This problem can be alleviated with synthetic aperture focusing technology using the coherence factor^9,33,34^. Second, the imaging speed is currently limited by the fixed 10 kHz PRF of our pulser-receiver. Third, global 3D volumetric imaging cannot be performed seamlessly due to motion artifacts between consecutive B-mode images (e.g., respiration and heartbeat). These problems can be overcome by image processing methods, such as compressive sensing, and electrocardiogram (ECG) gating technology^35–38^. In addition, the continuously steered mirror also could lead the motion artifact because of the directivity of acoustic. However, the mirror’s steering angle in the time gap while the acoustic beam travels from transmission to receiving was negligible in comparison with the numerical aperture of the transducer.

We believe that the MEMS-US-TT system has great potential for preclinical cardiac disease studies, where it can measure such cardiac performance parameters as the ejection fraction, stroke volume, and fractional shortening. In addition, the 3D volumetric imaging capability of this system can benefit oncology by showing an entire tumor’s shape. Moreover, ophthalmology widely uses ultrasound biomicroscopy with a high frequency US transducer.

We can easily transform this simple and compact MEMS scanner into another platform, MEMS-US-HH system, for *in vivo* human imaging. We demonstrated the real-time human vessel imaging capability and quantified the radial artery’s pulsation. Further, with a high PRF of the pulser-receiver, Doppler imaging and elastography will also be feasible^39^. Because our US imaging platform can be easily transformed, we can adapt any transducer to specific clinical condition. For example, a high frequency (20–30 MHz) US transducer will be used in US-guided radial artery catheterization and ophthalmology, and a low frequency (7.5–10 MHz) US transducer performs musculoskeletal imaging.

## Methods

### Detailed measurement and calculation processes of the main parameters

A customized single element ultrasound transducer used in this study has the aperture size of 9 mm, the focal distance of 21.8 mm, and the central frequency of 16.7 MHz. Lithuium niobate (LiNbO_3_) was used as the piezoelectric material of the transducer.

The length of the near field, N, is given by:

1 $$N={D}^{2}f/4c$$

where *D* is the element diameter, *f* is the frequency of the transducer, and *c* is the speed of sound. The length of focal zone, F_Z_, of this transducer is 4.1 mm, which is given by:

2 $${F}_{Z}=N\ast {(F/N)}^{2}[2/(1+0.5(F/N))]$$

where *F* is the focal length.

To measure spatial resolution of our system, we implemented B-scan imaging of a tungsten wire with a diameter of 50 μm (Supplementary Fig. S1(a)). We extracted the line pixels following lateral and axial direction, then fitted a line spread function (LSF). The full widths at half maximum (FWHM) of the LSFs were considered as the lateral and axial resolution, respectively (Supplementary Fig. S1(b,c)).

### Fabrication of a water-proofed MEMS scanner

We developed a water-proofed MEMS scanner to steer acoustic paths rapidly for real-time B-mode US imaging. The fabrication process is detailed in previous studies^11–13^. Briefly, the MEMS scanner has a mirror assembly and body assembly. The mirror itself is moved rapidly and precisely by electromagnetic force generated between permanent magnets in the mirror assembly and coils in the body assembly. The mirror assembly is composed of an acoustically reflective mirror, two neodymium magnets, and a polydimethylsiloxane (PDMS) layer with tongues that act as rotation hinges. The PDMS layer can be steered because it has low stiffness. The acoustic mirror is made from a silicon carbide wafer which has high acoustic impedance and hence high US reflectivity. This mirror is attached on the PDMS layer, and the two neodymium magnets are embedded in the PDMS layer. The body assembly is composed of an aluminum holder and two coils that are wrapped around steel rods and tightly embedded in the aluminum holder. The mirror assembly and the body assembly form one unit because the rotation hinges of the PDMS layer are adhered to the body part. The two coils in the body are closely aligned with the two neodymium magnets in the mirror assembly. When an AC voltage is applied to these two coils, an electromagnetic field is generated: the acoustic mirror is steered continuously by the attractive and repulsive forces between the magnets and coils. The water-proofed MEMS scanner used in this study has a scanning speed of 40 fps as the resonance frequency. This water-proofed MEMS scanner are mounted in a 3D printed fixture with the customized transducer. This fixture makes the angle between the acoustic path and the mirror 45° to sweep the acoustic path.

### *In vitro* phantom imaging

Ultrasonic images of a leaf skeleton were obtained by the MEMS-US-TT system. The leaf skeleton sample was fixed on a glass slide and positioned within a water tank. We defined the MEMS scanning direction as X, the elevational direction of the MEMS module as Y, and the depth direction as Z. One local volumetric dataset was acquired using the MEMS scanner and the Y-axis motorized stage, and it covered 10 mm × 25 mm × 6 mm along the X, Y, and Z axes, respectively. After the first local volumetric dataset was acquired, the X-axis motorized stage was moved in a 4 mm step, and the next local volumetric dataset was obtained. Finally, the six local datasets were merged into one global volumetric dataset. Therefore, the total imaging range was 30 mm × 25 mm × 6 mm along the X, Y, and Z axes, respectively.

### *In vivo* small animal imaging

*In vivo* small animal imaging was conducted using the MEMS-US-TT system, following regulations and guidelines approved by the Institutional Animal Care and Use Committee (IACUC) of Pohang University of Science and Technology (POSTECH). Healthy BALB/c mice (POSTECH Biotech Center) at an age of 6 to 9 weeks were used for the experiments. The mice were initially anesthetized with isoflurane (1 L/min of oxygen and 0.75% isoflurane) using a gas system (VIP 3000 Veterinary Vaporizer, Midmark, USA). Then, the hair was removed using an electric shaver and a depilatory. We applied US gel (Ecosonic, SANIPIA, Republic of Korea) to the animals, and then placed them in close contact with the outside of vinyl film on the bottom of the water tank.

The size of one cross-sectional B-mode image was 13 mm × 16 mm along the X and Z axes, respectively. The lateral length was defined as the maximum MEMS scanning range of 13 mm, and the axial depth was defined as 16 mm, which was affected by the focal length of the transducer and the pulse-receiver’s voltage. When we monitored dynamic movements, such as heartbeat, real-time B-mode imaging was implemented at 40 fps. The acoustic focus was located approximately 6 mm below the mouse skin for heart imaging, and 3 mm below for throat imaging.

When we acquired volumetric data on the mouse heart, one local volumetric dataset covered the 13 mm × 10 mm × 16 mm area (X, Y, and Z axes), and the X-axis motorized stage moved in 4 mm steps. Four local datasets with an acquisition time of 33 seconds for all four were acquired to form a large FOV global volumetric dataset. The post image processing (e.g., demodulation and scan conversion) took about 35 seconds, and the volume merging took about 15 seconds. For throat imaging, one local dataset covered the 13 mm × 15 mm × 16 mm area (X, Y, and Z axes), and nine local datasets were used to get one global volumetric image. It took the acquisition time of 80 seconds, the post image processing of 111 seconds, and the volume merging time of 32 seconds. The heart images measured 25 mm × 10 mm × 16 mm respectively (X, Y, and Z axes), and the throat images measured 29 mm × 15 mm × 16 mm (X, Y, and Z axes). We also could see a real-time B-mode image while acquiring 3D volumetric data.

### *In vivo* human imaging

*In vivo* human imaging was performed with the MEMS-US-HH system. All experimental procedures followed a clinical protocol approved by the Institutional Review Board (IRB) at POSTECH. A healthy volunteer gave fully informed consent for imaging of her wrist.

The experiment proceeded as follows. First, the palm of the volunteer was positioned pointing upwards, and US gel was applied to the wrist above the radial artery. Then, B-mode images were captured at 40 fps over a FOV measuring 13 mm × 16 mm (X and Z axes). To differentiate the radial artery and veins, we repeatedly compressed and relaxed the volunteer’s wrist with our system. Additionally, we monitored the pulsation of the radial artery.

## Supplementary information

Supplementary Figure S1: Lateral and axial spatial resolutions of the MEMS-US system.

Supplementary Movie S1: Volume rendering of the leaf skeleton.

Supplementary Movie S2: Volume rendering of the mouse heart.

Supplementary Movie S3: Volume rendering of the mouse throat.
