# Supplementary material for: Versatile Single-Element Ultrasound Imaging Platform using a Water-Proofed MEMS Scanner for Animals and Humans
Source: Sci Rep. 2020 Apr 16;10:6544. doi: 10.1038/s41598-020-63529-z (PMC7162865; doi:10.1038/s41598-020-63529-z)
Supplement: Supplementary file 1 — Supplementary Figure S1: Lateral and axial spatial resolutions of the MEMS-US system. [file 41598_2020_63529_MOESM1_ESM.docx]

**Supplementary Information**

**Versatile Single-Element Ultrasound Imaging Platform using a Water-Proofed MEMS Scanner for Animals and Humans**

**Seongwook Choi**^†^**, Jin Young Kim**^†^**, Hae Gyun Lim, Jin Woo Baik, Hyung Ham Kim*, and Chulhong Kim***

Department of Creative IT Engineering, Electrical Engineering, and Mechanical Engineering, Pohang University of Science and Technology (POSTECH), Pohang, 37673, Republic of Korea

**Supplementary Information Contents:**

- **Supplementary Figure S1:** Lateral and axial spatial resolutions of the MEMS-US system.
- **Supplementary Movie S1:** Volume rendering of the leaf skeleton
- **Supplementary Movie S2:** Volume rendering of the mouse heart
- **Supplementary Movie S3:** Volume rendering of the mouse throat


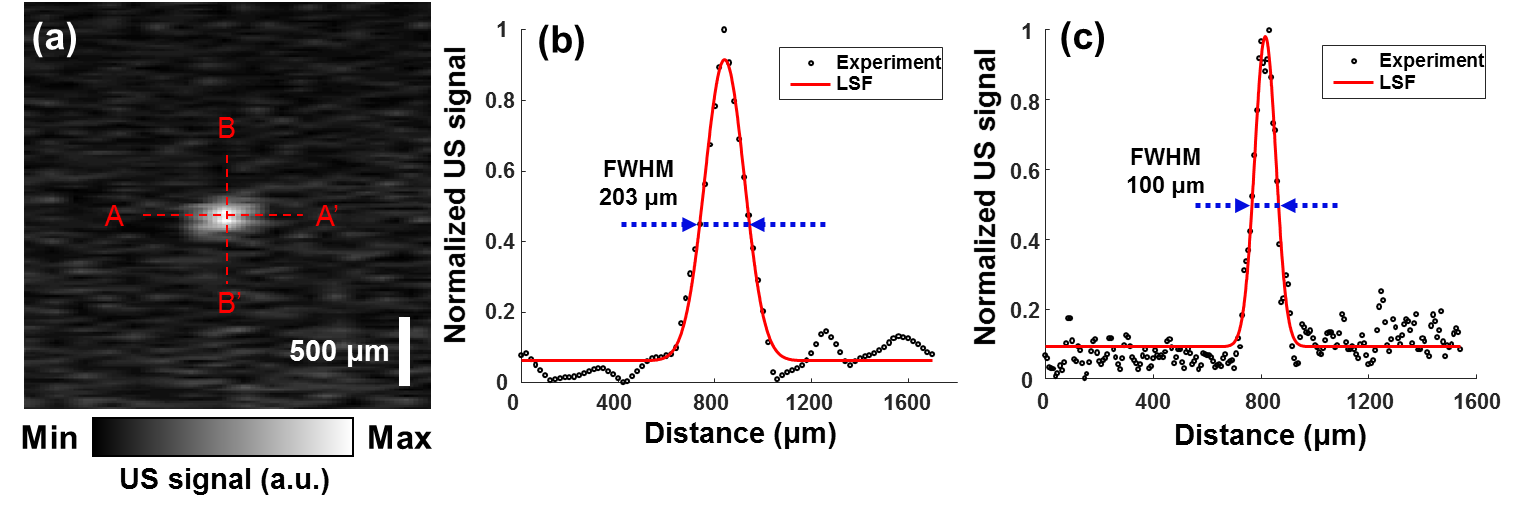


Supplementary Figure S1. Lateral and axial spatial resolutions of the MEMS-US system. (a) B-scan image of the tungsten wire. LSF fittings from the experimental data across (b) the A-A’ line and (c) the B-B’ line in (a). US, ultrasound; FWHM, full width at half maximum; LSF, line spread function.
